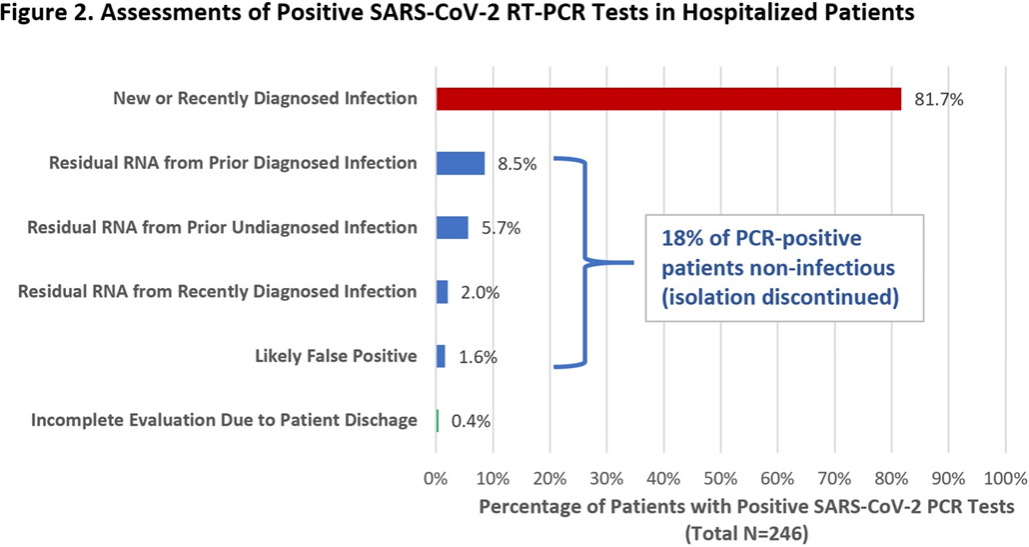# Does Every Patient with a Positive SARS-CoV-2 RT-PCR Test Require Isolation? A Prospective Analysis

**DOI:** 10.1017/ash.2021.15

**Published:** 2021-07-29

**Authors:** Chanu Rhee, Meghan Baker, Sanjat Kanjilal, Robert Tucker, Vineeta Vaidya, Amy Badwaik, Elizabeth Mermel Blaeser, Cassie Coughlin, Jennifer Elloyan, Candace Hsieh, Meghan Holtzman, Ofelia Solem, Michael Klompas

## Abstract

**Group Name:** CDC Prevention Epicenters Program **Background: Reverse**-transcriptase polymerase chain reaction (RT-PCR) tests are the reference standard for diagnosing SARS-CoV-2 infection, but false positives can occur and viral RNA may persist for weeks-to-months following recovery. Isolating such patients increases pressure on limited hospital resources and may impede care. Therefore, we quantified the percentage of patients who tested positive by RT-PCR yet were unlikely to be infectious and could be released from isolation. **Methods:** We prospectively identified all adults hospitalized at Brigham and Women’s Hospital (Boston, MA) who tested positive for SARS-CoV-2 by RT-PCR (primarily Hologic Panther Fusion or Cepheid Xpert platforms) between December 24, 2020, and January 24, 2021. Each case was assessed by infection control staff for possible discontinuation of isolation using an algorithm that incorporated the patient’s prior history of COVID-19, current symptoms, RT-PCR cycle threshold (Ct) values, repeat RT-PCR testing at least 24 hours later, and SARS-CoV-2 serologies (Figure 1). **Results:** Overall, 246 hospitalized patients (median age, 66 years [interquartile range, 50–74]; 131 [53.3%] male) tested positive for SARS-CoV-2 by RT-PCR during the study period. Of these, 201 (81.7%) were deemed new diagnoses of active disease on the basis of low Ct values and/or progressive symptoms. Moreover, 44 patients (17.9%) were deemed noninfectious: 35 (14.2%) had prior known resolved infections (n = 21) or unknown prior infection but positive serology (n = 14), high Ct values on initial testing, and negative or stably high Ct values on repeat testing. Also, 5 (2.0%) had recent infection but >10 days had passed since symptom onset and they were clinically improving. In addition, 4 (1.6%) results were deemed false positives based on lack of symptoms and at least 1 negative repeat RT-PCR test (Figure 2). One patient was asymptomatic with Ct value <35 but was discharged before further testing could be obtained. Among the 44 noninfectious patients, isolation was discontinued a median of 3 days (IQR, 2–4) after the first positive test. We did not identify any healthcare worker infections attributable to early discontinuation of isolation in these patients. **Conclusions:** During the winter COVID-19 second surge in Massachusetts, nearly 1 in 5 hospitalized patients who tested positive for SARS-CoV-2 by RT-PCR were deemed noninfectious and eligible for discontinuation of precautions. Most of these cases were consistent with residual RNA from prior known or undiagnosed infections. Active assessments of SARS-CoV-2 RT-PCR tests by infection control practitioners using clinical data, Ct values, repeat tests, and serologies can safely validate the release many patients from isolation and thereby conserve resources and facilitate patient care.

**Funding:** No

**Disclosures:** None

**Figure 1.**
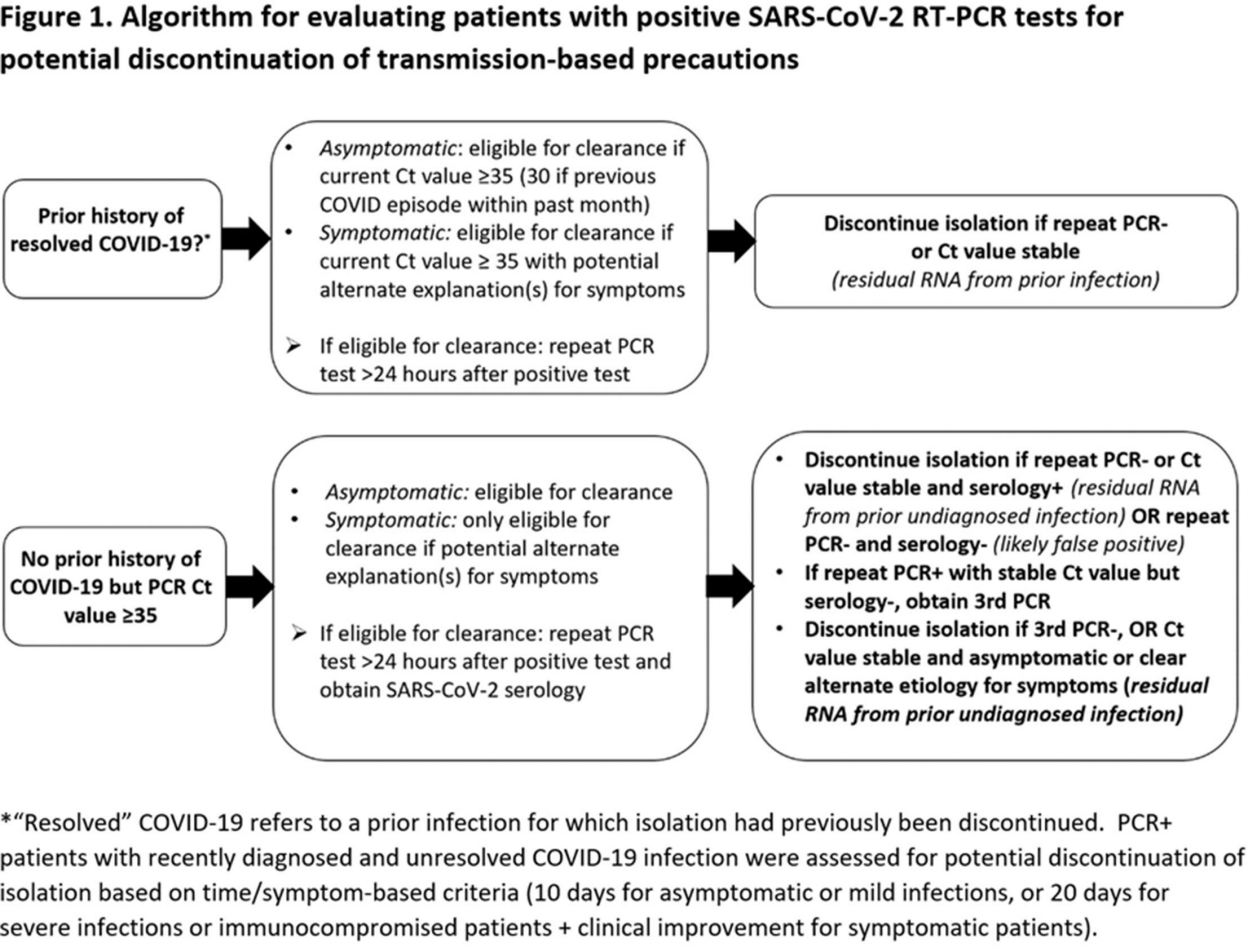

**Figure 2.**